# Melt-Flow Behaviours of Thermoplastic Materials under Fire Conditions: Recent Experimental Studies and Some Theoretical Approaches 

**DOI:** 10.3390/ma8125492

**Published:** 2015-12-15

**Authors:** Paul Joseph, Svetlana Tretsiakova-McNally

**Affiliations:** 1 Centre for Environmental Safety and Risk Engineering, Victoria University, PO Box 14428, Melbourne, VIC 8001, Australia; paul.joseph@vu.edu.au; 2 The Built Environment Research Institute, School of the Built Environment, Ulster University, Newtownabbey BT37 0QB, UK

**Keywords:** thermoplastics, thermal decomposition, flammability, melt-flow behaviour

## Abstract

Polymeric materials often exhibit complex combustion behaviours encompassing several stages and involving solid phase, gas phase and interphase. A wide range of qualitative, semi-quantitative and quantitative testing techniques are currently available, both at the laboratory scale and for commercial purposes, for evaluating the decomposition and combustion behaviours of polymeric materials. They include, but are not limited to, techniques such as: thermo-gravimetric analysis (TGA), oxygen bomb calorimetry, limiting oxygen index measurements (LOI), Underwriters Laboratory 94 (UL-94) tests, cone calorimetry, *etc.* However, none of the above mentioned techniques are capable of quantitatively deciphering the underpinning physiochemical processes leading to the melt flow behaviour of thermoplastics. Melt-flow of polymeric materials can constitute a serious secondary hazard in fire scenarios, for example, if they are present as component parts of a ceiling in an enclosure. In recent years, more quantitative attempts to measure the mass loss and melt-drip behaviour of some commercially important chain- and step-growth polymers have been accomplished. The present article focuses, primarily, on the experimental and some theoretical aspects of melt-flow behaviours of thermoplastics under heat/fire conditions.

## 1. Introduction

Materials made of thermoplastic polymers such as polyethylene (PE), polypropylene (PP), poly(methyl methacrylate) (PMMA), polyacrylonitrile (PAN), polystyrene (PS), polyamide (PA-6), polycarbonate (PC), polyvinyl chloride (PVC), polyurethane (PU), *etc.* are widely available on the market, and are increasingly being used in a range of commercial products, from textiles to moulded items. However, these materials are highly flammable and for their safe and wider usage they need to be sufficiently flame retarded, either using appropriate additive(s), or through adequate levels of chemical modification(s). The flammability and resulting destruction of property, and the detrimental effects on the environment, are not the only major problems. Fire fatalities are predominantly caused by the evolved smoke and toxic gases, exacerbated in some cases by poisonous fumes emitted from synthetic organic polymers [1]. In addition, many thermoplastic materials tend to melt-flow and melt-drip under heat/fire, and this can pose a very serious secondary hazard in fires involving them—for instance, in the situations where polymeric materials are used in the construction of doors, windows, ceilings and roofs, curtains, *etc.* It is believed that in the Stardust night club fire (1981, Dublin, Ireland), which led to 48 fatalities and 214 serious injuries [2], one of the main reasons responsible for the escalation of the fire was that the melt-flow droplets from the polymeric lining of the ceiling caused ignition of the seat-cushions, which were made of polyurethane.

Generally, the combustion of a polymeric material is a highly complex process, which involves a series of interrelated and/or independent stages occurring both in the condensed phase and in the gaseous phase, or at the interphase between these two. Under the influence of heat/fire, thermoplastic polymeric materials often undergo deformations, and this will lead to corresponding drastic changes in their mechanical and associated properties [3]. In addition, the effect of melt-flow, melt-drip behaviour and secondary ignition of other possible fuel loads in the vicinity can complicate the situation further and can also lead to the escalation of fire. 

Currently, a range of qualitative, semi-quantitative and quantitative testing techniques are employed, both at laboratory scale and for commercial purposes, to evaluate the complex behaviour of polymeric materials that are exposed to heat and/or fire. They include, but are not limited to, techniques such as: thermo-gravimetric analysis (TGA), oxygen bomb calorimetry, limiting oxygen index (LOI) measurements, Underwriters Laboratory 94 (UL-94) tests, cone calorimetry, *etc.* All of the above mentioned methods have their own advantages and disadvantages. For example, the UL-94 test which is commonly used as an industrial standard, is a vertical burning test of a solid plaque of the material, where an ignition source is applied to the bottom tip of the specimen hung in a chamber of ambient air. In this test, a specimen (125 mm × 12.5 mm × (3–4) mm in size) is placed above a Bunsen burner with a 20 mm flame height. Initially, the flame is applied to a polymer sample for 10 s and then removed. If the flame on the tested sample is extinguished, the burner is applied again for 10 s. The flaming times, any occurrence of dripping, and a propensity of melted drops to ignite surgical cotton placed underneath the sample, are usually observed and recorded in the test runs. Generally, the test leads to a pass/fail criterion of the material in terms of classifications, such as: V-0, V-1, V-2, *etc*. Here, a V-0 rating is considered as a “pass” (burning stops within 10 s on a vertical specimen; drips of particles allowed as long as they are not inflamed), whereas V-1 (burning stops within 30 s on a vertical specimen; drips of particles allowed as long as they are not inflamed) and V-2 (burning stops within 30 s on a vertical specimen; drips of flaming particles are allowed) are considered as a “fail” in the test.

When objects containing thermoplastic polymers are exposed to heat and/or a fire, they usually exhibit a melt-flow and/or dripping behaviour. As a result of a polymer’s melt-flow behaviour, effected downwards under the influence of the gravitational force, two scenarios are possible: either further burning ceases owing to the removal of mass and heat from the primary pyrolysis zone (safe route), or it could become a secondary source of ignition, which, in turn, could lead to the acceleration of the growth of the fire (hazardous route). The latter case occurs when the polymer droplets either constitute a pool of fuel for further burning or cause secondary ignition of other combustible materials that they come in contact with. Typically, when the liquid pool is ignited, the burning surface area increases drastically and the growing pool fire could ignite neighbouring objects, thus resulting in an increased overall burning rate and heat release rate; the thermal feedback from this could then result in the enhanced production of a polymer melt. If the pool fire is not contained, this may potentially lead to the spreading of the fire along the horizontal surfaces (e.g., flooring) [4]. Generally, the following factors can affect the development of the overall hazardous scenario: size of the burning object, chemical nature of the materials and surface area below the object, size and the temperature of melting drops, and frequency of dripping (*i.e.*, number of drops formed in unit time). 

The melt-drip behaviour can be explained by a combination of physical factors that accompany the thermal/chemical decomposition pathways of a polymer [5]. Thermoplastic polymers often will soften and flow when they are heated to temperatures exceeding their glass transition temperature, melting point, or viscous flow temperature. Generally, melting under the influence of an external heat source will result in the polymeric chains gaining a higher degree of translational mobility. Another factor that should be considered here is the concomitant reduction in the viscosity of the melt, which also increases as the thermal decomposition of polymeric chains leads to the formation of smaller fragments (oligomers) and/or low molecular weight species. Zhang *et al.* found that the degree of melt-flow is directly proportional to the glass transition temperature (*T*_g_) of a polymer: *i.e.*, polymers with the lower glass transition temperatures melt-drip significantly more than those with higher values [6]. 

The melt-flow/drip phenomenon among polymers, although reported widely, is yet to be fully understood. This problem still needs a rigorous quantitative approach in establishing the factors affecting melt-dripping behaviour of different thermoplastics. Although there are many tests available to measure the flammability of polymeric materials, only the UL-94 vertical burning test allows, at least, a qualitative evaluation of their melt-flow and melt-drip behaviours. Unfortunately, as mentioned earlier, this technique only yields qualitative information [5,7].

## 2. Experimental Approaches

Many research groups have attempted to quantitatively assess the melt-flow and melt-drip behaviours of polymeric materials [5,8,9,10,11,12,13,14]. The vast majority of these studies were based on the UL-94 experiments. Most of these research groups also suggested methodologies that were based on measurements of the mass of drops collected and weighted after the testing of vertically oriented polymer samples. For example, the size and the mass of the drops formed during the UL-94 test were measured in the study carried out on PA-6 systems (BASF, Florham Park, NJ, USA) [10,12,15]. Other studies, performed by different research groups [4,5,8,9,11,13,14,16] were focused on the evaluation of polymers’ melt-flow/drip behaviour in fires, whilst those carried out at the University of Bolton, UK, also considered non-flaming operating conditions [7,17,18,19]. The parameters measured and reported in the literature include: numbers, masses, shapes, sizes of individual drops [7,10,12,15]; real-time mass data and mass loss rate [5,13,14]; dripping time and the time at which the first drop appears and falls down [5,7,17,19]; and the viscosity of melts [18,19].

**Figure 1:**
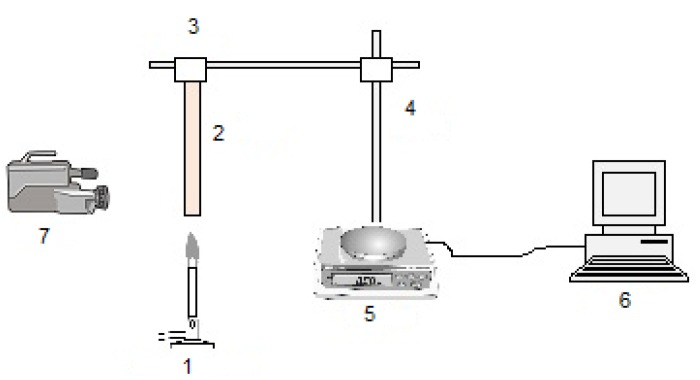
A schematic sketch of the experimental set-up for the UL-94 vertical test: 1—Bunsen burner; 2—tested polymer sample; 3—clamp; 4—bracket; 5—balance; 6—computer; 7—camera (used by Wang *et al.* [5,13]).

Wang *et al.* [5,13] have studied the burning and dripping behaviours of eight polymeric materials under the UL-94 vertical test conditions. The polymeric systems tested included: acrylonitrile butadiene styrene (ABS), low-density polyethylene (LD-PE), PA-6, PC, PMMA, PP, PS and white pine. The experimental set-up used in this work is schematically shown in Figure 1. A polymeric sample (2), attached to a clamp (3), was orientated vertically above the flame from a Bunsen burner (1). A clamp was fixed to a bracket (4), which was then placed on an analytical balance (5). The real-time mass data (*i.e.*, mass retention/mass loss) were recorded every second with the aid of a computer (6) connected to the balance. A camera (7) was employed for the entire duration of the testing to record the burning and dripping phenomena of the polymer in question. The experiments were carried out in a quiescent air environment. 

Wang *et al.* also reported two modes of melt-drip phenomena: a wax-like dripping with smaller sized polymeric drops for samples of PP, LD-PE and PA-6, and another mode associated with larger sized drops, characteristic for ABS, PC, PMMA and PS [5]. It should be noted here that these two types of melt-dripping were gauged by the size of the polymeric specimen before and after the dripping, rather than by the actual diameter of the drops. It was also observed that the melt-dripping of ABS, PMMA and PS happened relatively later (for example, for 2 mm thick samples, the first dripping occurred at 65–66 s for ABS, at 61–62 s for PMMA, and at 22–23 s for PS) during the experimental run and led to a significant mass loss, whereas LD-PE and PP began to melt-drip earlier on (for 2 mm thick samples time of first dripping for LD-PE was recorded at 13–14 s and for PP—at 12–13 s) and often with drops having relatively lower masses [5]. In addition, the authors observed that the melt-flow of PA-6 and PC led to flame extinction, and there was no dripping for a white pine sample due to a char formation. 

During the course of their investigation, Wang *et al.* found that the mass loss of tested polymeric samples was within 0.001–0.01 g/s range [5]. In addition, it was reported that the mass reduction, before the flame reached the clamp and the polymer began to drip, only accounted for a small fraction of the initial mass of the specimen, which did not exceed 4 wt %. Thus, it was concluded that the UL-94 rating depended on the burning of a relatively small portion of the polymer specimen. For the polymers tested by the authors, the mass of the first drop was found to be in the range of 0.0012 to 0.9045 g, while the diameter of the first drop was within 2.0–10.0 mm range. The mass of the first drop also increased with the time taken for it to form and fall under gravity. It was concluded that the dripping rates decreased when the size and mass of the drops increased in a typical UL-94 vertical burning test [5]. 

**Figure 2:**
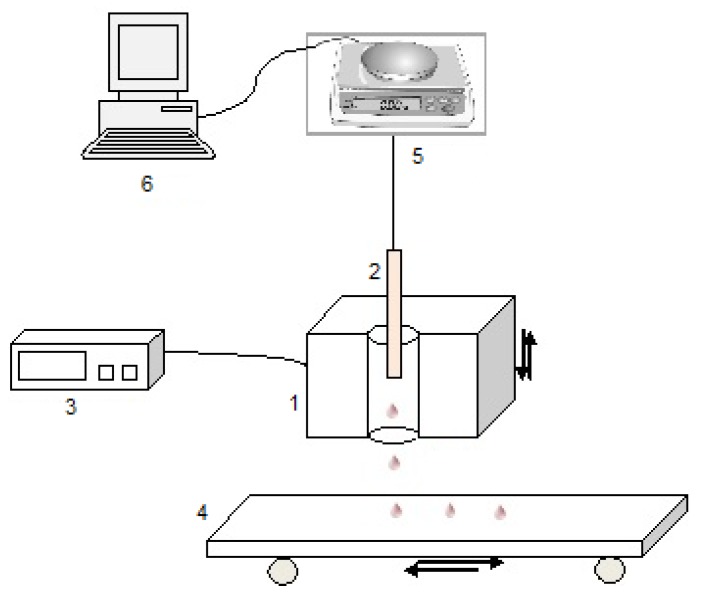
A schematic sketch of the experimental set-up to study the melt-drip phenomenon in non-flaming conditions: 1—electric mobile furnace; 2—tested polymer sample; 3—adjustable temperature controller; 4—conveyer belt with aluminium foil; 5—balance; 6—computer (used by [7]).

**Figure 3:**
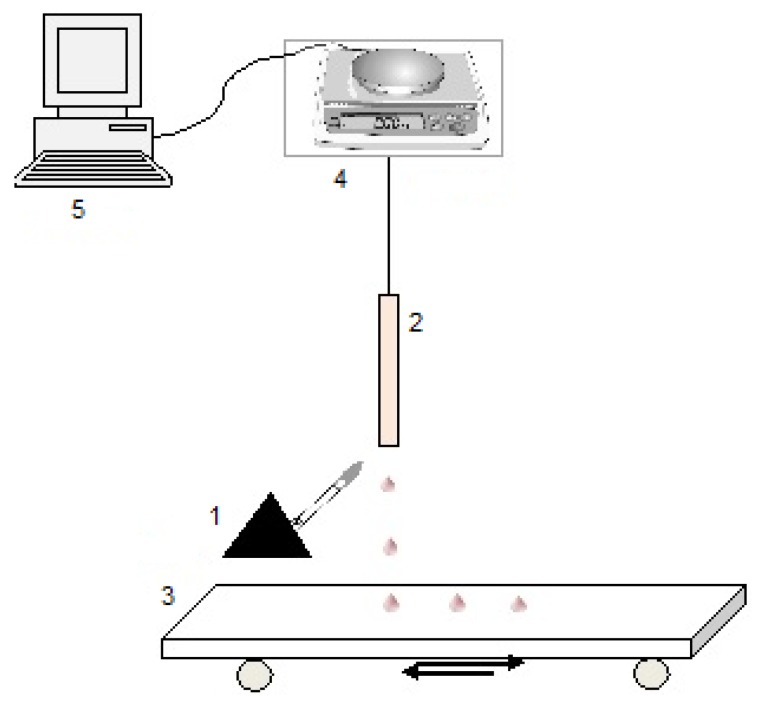
A schematic sketch of the experimental set-up equivalent to the UL-94 test: 1—burner; 2—tested polymer sample; 3—conveyer belt with aluminium foil; 4—analytical balance; 5—computer (used by Kandola *et al.* [7]).

The research group at the University of Bolton, UK, has developed an experimental technique, which allowed them to quantitatively evaluate the melt-flow/drip behaviour of thermoplastic polymers in real time. They reported on the melt-drip behaviours of PP, PA-6, PMMA, PS, PC and polyethylene terephthalate (PET) exposed to a radiative heat flux imposed by a purpose-built furnace [7,17,18,19]. For the purpose of comparison, the authors also conducted experiments that replicated the flaming conditions of the UL-94 vertical test. The schematic diagram of the experimental rig used by them is shown in Figure 2 and Figure 3. The polymer samples were prepared as plaques of two different sizes: “small size” 100 mm × 6 mm × (3–4) mm and “standard size” 125 mm × 12.5 mm × (3–4) mm. 

As is shown in Figure 2, a polymer sample (2) was placed in an 800 W electric furnace (1) that was previously heated to a set temperature. The furnace had a tube-like borehole of dimensions: 120 mm long and 25 mm in diameter. The temperature in the furnace was adjusted with a temperature controller (3), which could measure the core surface temperature with the aid of a thermocouple. The polymeric sample was attached through a thin wire and a built-in hook to the bottom of a balance (5), which in turn was connected to a computer (6). The mass loss of the sample was recorded in real time by using appropriate data acquisition software. The furnace, equipped with a pulley mechanism, was able to move up or down until the bottom tip of the sample was located at the centre of the furnace bore. The thermocouple was inserted in a tube orifice in order to measure the air temperature inside the bore. A conveyer belt (4), moving back and forth at a constant rate of 11.2 cm/s, with a pre-weighed aluminium foil strip, was placed underneath the furnace borehole to collected the melt drops of the polymeric sample. By weighing the foil, before and after the test, the total mass of drops produced can be evaluated. In addition to this, the number, size (diameter and thickness), shape and distance (*i.e*., time) between individual drops were evaluated by taking pictures of the aluminium foil during and after the experiments [7]. 

Each polymer tested at the University of Bolton, UK, was characterised by a specific temperature interval *T*_D–I_, between the D-point, when sample began to melt-drip, and I-point, when it ignited and burned. Four temperature values were selected by the authors from the *T*_D–I_ interval to set the furnace temperature for each type of polymer. From the curves of mass retention (measured as percentages) *versus* time (in seconds), obtained through the experiments, the following parameters were evaluated: time to the first melt drip, mass of the first drop, total number and mass of the melt drops. The mass loss recorded on the balance was associated with volatilisation and melt-dripping of the polymeric sample. The degree of volatilisation was subsequently calculated by subtracting the total mass of drops collected on the aluminium foil from the total mass loss.

As mentioned earlier, all the experiments were repeated by Kandola *et al.* in the experimental set-up without the furnace, *i.e.*, in the flaming conditions similar to the vertical UL-94 test [7]. As seen from the schematic sketch, shown in Figure 3, a sample of a polymer (2) hung on a hook was attached to a balance (4) connected to a computer (5) for mass data acquisition. A bottom-end of the tested polymeric sample was placed above the flame (20 mm high) from a Bunsen burner (1) as per the standard UL-94 procedure. The drops produced during melting and burning of the sample were collected on a foil strip, placed on a conveyer belt (3) (Figure 3). 

In addition to the above-mentioned experiments, the researchers also performed thermo-gravimetric and associated analyses (TGA/Derivative Thermogravimetric Analysis (DTG)/Differential Thermal Analysis (DTA)) of polymers and their droplets in air and nitrogen atmospheres. Based on the TGA data obtained in air for the polymers and droplets, the extent of decomposition (as a percentage) was calculated [7]. The summary of the data reported by Kandola *et al.* [7] including glass transition temperature *T*_g_, *T*_D–I_ interval, time range corresponding to first melt dripping, lower and higher values of masses and diameters of individual drops, total number of drops collected, as well as calculated values of degree of volatilisation and degree of decomposition in air and in nitrogen atmospheres is given in Table 1. 

**Table 1:** Summary of melt dripping data obtained by Kandola *et al.* [7].

Melt Dripping Data	Polymers					
	PP	PA-6	PC	PET	PS	PMMA
*T*_g_ (°C)	−26	54	147	68	96	110
*T*_D–I_ (°C)	118	223	228	237	75	100
Time to First Melt Drip (s)	6–9	9–32	6–19	6–28	9–15	0–50
Mass of an Individual Drop (mg)	3–6	20–250	59–71	24–165	20–100	14–32
Diameter of an Individual Drop (mm)	4–6	11–17	11–12	9–11	9–10	4–5
Number of Drops	54–90	2–10	7–12	7–12	13–20	10–14
Degree of Volatilisation (%)	33–42	32–40	21–25	16–32	14–22	42–67
Degree of Decomposition in Air (%)	36.8	26.6	6.0	−1.2	26.0	35.0
Degree of Decomposition in Nitrogen (%)	49.0	13.2	3.0	−4.6	7.4	−13

PP: polypropylene; PA-6: polyamide; PC: polycarbonate; PET: polyethylene terephthalate; PS: polystyrene; PMMA: poly(methyl methacrylate).

## 3. Mechanistic Aspects

The melt-flow/drip behaviour of polymers depends on the underpinning mechanism(s) of their pathways of thermal decomposition. Correlations between the mechanism of polymer decomposition and its melting/dripping pattern were suggested by many researchers [5,7,17,18,19]. 

The three most common schemes for polymer decomposition mechanism are [3]:

1. A random chain cleavage followed by further chain scission is characterised by low monomer yields in decomposition products and a rapid drop in molecular weight (e.g., for PE, PP).
2. A random chain cleavage followed by chain unzipping is characterised by high monomer yields in the decomposition products and a slow decrease in molecular weight of the polymer (e.g., for PMMA, poly(α-methyl)styrene, polytetrafluoroethylene, *etc.*).
3. An intra-chain chemical reaction followed by cross-linking reaction and carbonaceous residue formation, or random chain cleavage. This process generates a relatively high yield of volatiles from the inter-chain reaction, produces very little monomer, and accompanied with no or only a very slight decrease in molecular weight during the initial stages of decomposition (e.g., for PAN, PVC, *etc.*).

In some cases, several of the above mentioned schemes may occur simultaneously, depending on the sample size, heating rate, pyrolysis temperature, environment, and presence of any additives. 

Wang *et al.* attempted to correlate the melt-drip behaviour with the mechanism of polymer decomposition under the influence of heat/fire [5]. For polymers such as PP and PE, decomposing via random chain scission, small-sized drops were found to occur. For polymers such as PMMA, which degrade mainly by chain unzipping reaction, large-sized drops are likely to appear in vertical burning tests like the UL-94. The results obtained by Kandola *et al.* were in conformance with the established routes of polymer decomposition—*i.e.*, PMMA will undergo predominantly end-chain scissions, resulting in near quantitative yield of the monomer, while PP, PA-6, PC, PET and PS will degrade mainly via random chain scissions [7]. 

The work carried out at the University of Bolton, UK, also demonstrated that the number, shape and the size of the individual melted drop was measured with good reproducibility [7]. These attributes associated with the melt-flow/drip behaviour actually depend on the chemical nature (*i.e*., depending on individual chemical classes, such as polyolefins, styrenics, acrylics, polyesters, polyamides, *etc.*) of the polymer in question and on the mechanism of its decomposition. The authors have classified the polymers they have tested in four groups.

Group I included PP that melt-drips rapidly, producing wax-like small drops (weighing from 3 to 6 mg and less than 6 mm in diameter), the number of which usually exceeds 50. It was characterised by the thermal range *T*_D–I_ = 118 °C. The degree of volatilisation for PP was in the range of 33%–42% and extent of decomposition in air was around 36.8%. The wax-like melt-drip behaviour of PP is explained in part by its low glass transition temperature (*ca.* −26 °C). Due to random chain cleavage, the thermal decomposition of PP leads to the production of shorter chains having lower viscosity, which, in turn, will increase its tendency to melt-flow and melt-drip. It was also found that the furnace temperature had no effect on the size of the drops, but the number of the drops increased as the temperature was raised. The TGA traces recorded for PP and its melted drops differed significantly, implying the significant degree of thermal decomposition (up to about 49%) in nitrogen atmosphere [7]. 

Group II included three polymers, PA-6, PET and PC, and the *T*_D–I_ range of these materials was higher than 220 °C. The curves of mass loss had distinct steps, with each one of them corresponding to melt dripping. The formation of large drops, with their mass ranging between 20 and 250 mg and diameter in the interval of 10–17 mm, was observed for this group of polymers. The number of collected drops on average was also found to be less than 12. For PA-6, the number of drops increased with the increase of temperature, but at the same time the mass of each drop decreased. The degree of volatilisation was observed to be decreasing in the following order: 32%–40% for PA-6 > 21%–25% for PC > 16%–32% for PET. There was no decomposition recorded for PET, while for PA-6 it was around 26.6% and for PC: 6.0%. The thermal decomposition of PA-6 occurs through random chain cleavages at the amide groups, leading to the formation of gaseous compounds such as NH_3_, CO and CO_2_ as well as low-molecular fragments. PA-6 can also undergo depolymerisation producing its cyclic volatile monomer, caprolactam. Therefore, the data obtained for this polymer varied significantly from one sample to another. The thermal decomposition of PET leads to an increased production of acetaldehyde and other gases (ethane, CO, CO_2_, *etc.*), whereas PC generates carbon dioxide and phenolic compounds. It was also noticed that the volatilisation rate for PET was doubled when the temperature increased from 415 to 635 °C, indicating that the degree of decomposition substantially increases with temperature. Interestingly, there was no difference in TGA results of PET and its drops, implying that PET displayed only melting without any noticeable thermal decomposition. For PC, unlike for PA-6 and PP, the rise in temperature had no effect on the number, sizes, or masses of the drops [7].

PS was allocated to Group III as its melt dripping in the furnace was noticeably different compared to other polymers studied. The temperature interval between the first melt drop and ignition, *T*_D–I_, was equal to 75 °C. The number of medium-sized drops was between 13 and 20. They were around 100 mm in diameter and their mass varied from 20 to 100 mg. The thermal decomposition of PS is dominated by random chain cleavage of the main carbon backbone, resulting in the formation of large oligomers, which may break down further to produce monomer, styrene, as well as the dimers and trimers. The PS decomposition in air was at approximately 26%, while in the nitrogen atmosphere was only 7.4%. It was also reported that the temperature increase inside the furnace did not affect the diameters of PS drops and the extent of volatilisation, although the number of drops tend to increase [7]. 

Group IV included PMMA, and its behaviour differed substantially from the other polymers. PMMA’s temperature range between first dripping and igniting was about 100 °C. The principle difference in the melt-drip behaviour of PMMA is that the volatilisation occurred prior to the melt dripping stage. PMMA’s melt dripping was also characterised by the production of 10–14 small drops (with the mass of each lower than 25 mg, and diameter around 5 mm). PMMA is known to degrade via random chain scission followed by chain unzipping generating between 90% and 100% of the monomers. This was reflected in the degree of volatilisation, above 40%, the highest value among all the polymers tested in the furnace. Furthermore, the formation of volatiles increased as the temperature in the furnace was increased. It was also observed that the temperature does not affect diameters and thicknesses of the melted drops. The TGA data obtained in nitrogen atmosphere for PMMA and its molten drops were similar, indicating little changes in the molecular weights or its distribution for the polymer chains in the drops compared to the virgin polymer [7].

The effect of the external temperature on the mass loss during the melt-flow/drip was also reported in the literature [7]. In general, the higher the temperature in the furnace, the earlier the volatilisation and melt-dripping commenced. The degree of volatilisation is increased, as the external temperature rises for PET and PMMA, which can be explained by the thermal decomposition mechanism of these polymers, where depolymerisation and chain unzipping dominate. It should be mentioned here that in the experiments with the furnace, the melt-dripping was caused only by heat, leading to a gradual decline in the mass of the sample. As for tests equivalent to the UL-94, when the polymer burns and generates more heat, its thermal decomposition occurred faster, thus leading to more rapid mass loss associated with the increased production of flammable volatiles and extent of melting. It was observed from the UL-94 equivalent experiments conducted in [7] that the samples of PP, PA-6, PS and PMMA were burnt completely, while PET and PC samples did not burn to a zero residual mass. The burning of PET was accompanied by the formation of drops, which took the flame away from the sample, causing it to self-extinguish. It was also concluded that, as the melt-dripping rate reduces, the burning rate increases.

Another critical parameter for the melt-flow/drip of polymers is the viscosity, which depends on the temperature and the molecular weight. The rheological studies carried out in the 170–380 °C temperature interval indicated that the dependence of complex viscosity on temperature for PET, PA-6, PS, PMMA and PC is non-linear; it decreases and reaches a minimum, and then increases again upon heating. It was also shown that the rate of dripping (*i.e*., the number of drops per second) did not depend on the complex viscosity of the melt only, and other factors can also influence the intensity of the melt-dripping [7]. The rheological studies also indicated that the viscosity of the melted drips was significantly lower than that for the virgin polymers, once again confirming that melt-flow arises as a result of the combination of physical melting and partial polymer decomposition. The viscosity is usually affected by the additives or fire retardants (FRs), altering the melt-drip behaviour of thermoplastics. Some FRs have no effect on the rheological properties of polymers and hence will have minimum or no effect on the melt-flow/drip. Other FRs, such as char-promoting melamine phosphate added to PP, lead to an increase in complex viscosity at temperatures above 250 °C and can either reduce or stop the melt-dripping [19]. Thus, a careful choice of FRs might enable one to modify the rheological behaviour, processability and fire performance of thermoplastics [20]. For example, PP in the presence of a nanoclay additive, has been shown to have a lower number of melted drops but formed larger sized ones [19]. 

One of the factors directly linked to a degree of thermal decomposition is the temperature of the melted drops. Several experimental and modelling techniques are reported to evaluate the surface temperatures of the polymeric samples tested and the temperatures of melting drops produced by melting polymers [9,18,21]. 

## 4. Theoretical Considerations 

Understandably, the first port of call to try to quantify, or attempt to model, the rather complex phenomenon of the melt-flow behaviour of a polymeric sample is the UL-94 test as described by various authors [5,18,21]. Previously, several researchers have also approached modelling of the problem through validating the results obtained through proprietary, in-house built experimental instrumentation [6,22]. In this Section, brief accounts of some early theoretical studies are first described, followed by some recent developments in the subject area. 

Butler *et al.* from the National Institute of Standards and Technology (NIST) have done pioneering work on a combination of experimental and modelling aspects of polymer melt-flow behaviours [22]. In the course of their investigation, the dripping behaviour of several types of PP was studied, by employing an in-house built apparatus where the polymeric sample was mounted vertically and exposed to a uniform radiant heat on one face from a cone heater placed on its side. The measurements included: mass loss from the sample, mass collected in a catch pan, surface temperature, and surface velocities. There was also an experimental provision for measuring and extrapolating the viscosity to higher temperatures where formation of bubbles in the polymer makes the standard rheometric measurements impossible. The kinetic parameters, used to model the gasification of the polymeric samples, were obtained from thermo-gravimetric runs performed at dynamic conditions. The thermal conductivity of the polymers was also measured using a standard commercial apparatus based on transient heating for the temperature range from 40 through 265 °C. The authors successfully employed a finite volume model to validate the experimental observations that used the volume of the method to tackle the highly distorted interface for the melting and dripping polymeric sample. The model also took into account the heat flux to the distorted interface, empirical viscosity as a function of temperature, flow due to gravity, and gasification.

Given that the computational modelling and simulation of the burning, melt-flow and flame spread of thermoplastics are extremely complex phenomena, the standard finite element method (FEM) was combined with concepts from particle-based techniques to form the particle finite element method (PFEM) [23,24,25]. PFEM method tries to capture the extremely complex scenario involving fluid flow, heat transfer, material decomposition, flame chemistry, surface tension, and the drastic changes in the shape of the sample subjected to melt flow. The PFEM is a powerful Lagrangian technique for modelling and analysis of complex multidisciplinary problems in fluid and solid mechanics involving coupled thermal effects, fragmentation and separation of fluid particles and fluid-particle interaction effects, among others. In PFEM, the particles represent the nodes of a finite element mesh, which can move freely according to the velocity field, transporting their momentum and physical properties. The authors were able to demonstrate the potential of the PFEM to model the drastic change of shape of polymer objects as they burn, melt and spread in the underlying floor, including self-contained situations.

Kandola *et al.*, in the course of their recent experimental investigations into the melt-flow behaviours of some commercially important thermoplastics, have also attempted quantifying the degradation and melt-dripping of polymers [18]. Here they have developed a simple, one-dimensional heat transfer model with a view to computing the surface temperatures of the polymer sample, at various experimental furnace temperatures, thus measuring in effect the temperature of the molten drops dripping from the melting surface. The model has then been validated against the experimentally determined temperatures. The temperatures of the molten drops, in turn, were found to aid in predicting the degree of decomposition in a polymer during its melt dripping. The latter attribute could be calculated from the kinetic analyses of the thermo-gravimetric curves obtained for both the polymeric material and its drops [18]. 

## 5. Conclusions

From an overview of the recent and pertinent literature precedents on the melt-dripping behaviour of thermoplastics, it is evident that the exact physio-chemical processes underpinning the causes, the actual courses and the full consequences of a polymer decomposition are far from fully understood. However, the hazardous implications of such behaviours from moulded thermoplastic materials and, to some extent, thermosets have proved to be very severe. This arises not only from the point of view of their degree of contribution of the molten polymer as a primary fuel, but also owing to their potential in propagating and enhancing the severity of fires. Needless to say, the changes in the rheological properties that dictate the variations in melt-flow behaviours of thermoplastics, are often complicated by thermal and thermo-oxidative decomposition occurring in the melted polymer and through the formation of bubbles. This, in addition to the changes in the original geometry of the moulded polymeric material, makes the modelling attempts even harder. The situations could get even more complicated if other additives, such as plasticizers, anti-oxidants and flame retardants are present in the polymeric finished products [20]. Therefore, a more concerted effort is needed in both experimental and theoretical aspects of the problem before we can fully understand, and subsequently alleviate the problems arising from, the melt-flow behaviours of commonly-used moulded thermoplastic materials under the effects of heat/fire. 
